# Standardising infection control in CT intravenous contrast practice: an eDelphi study

**DOI:** 10.1186/s13244-026-02360-y

**Published:** 2026-07-29

**Authors:** Suzanne Hill, Yobelli Alexandra Jimenez, Sarah Lewis, Dania Abu Awwad

**Affiliations:** 1 Discipline of Medical Imaging Sciences, Sydney School of Health Sciences, Faculty of Medicine and Health, The University of Sydney, Sydney, NSW Australia; 2 Campbelltown Campus on Dharawal Country, Western Sydney University, Penrith, NSW Australia

**Keywords:** Computed tomography, Infection prevention and control, Intravenous contrast

## Abstract

**Objectives:**

Develop consensus-based recommendations for infection, prevention and control (IPC) practices in CT regarding (1) guiding principles for Intravenous (IV) Contrast training and compliance and (2) best practices for the use of IV contrast.

**Materials and methods:**

A modified e-Delphi study was conducted with a panel of experienced CT radiographers and nurses from healthcare settings across Australia. Nineteen statements were presented to participants who selected a response on a 5-point Likert scale from ‘strongly agree’ to ‘strongly disagree’ using an online survey. Participants were invited to provide justifications regarding their selected responses. Consensus was defined as ≥ 75% agreement, and any statement that fell below this benchmark was modified for subsequent rounds based on participants’ comments.

**Results:**

Three rounds were conducted, with the number of participants per round being 34, 26 and 24, respectively. Eighteen statements reached consensus, and pertained to guiding principles for IV contrast, sequencing IV contrast administration, single- and multi-use IV contrast administration systems and IPC considerations in the CT environment. Statements that failed to reach consensus (*n* = 1) or lost stability across subsequent rounds (*n* = 3) related to dripped contrast and connection/disconnection of the contrast tubing to the patient.

**Conclusion:**

This study established a set of eighteen IPC Guiding Principles and Best Practice statements for staff administering IV contrast in CT departments in the Australian healthcare setting. Findings underscore the importance of standardising IPC training and clearer communication of IPC policies. These results can inform policy updates and guide future education.

**Key Points:**

- **Question:** CT IPC practices vary, with no clear, relevant guidelines.
- **Findings:** Eighteen consensus statements guide CT IPC practices for IV contrast administration. Expert-led consensus statements enhance CT IV contrast practice, supporting safer departmental procedures.
- **Critical relevance statement:** CT IV contrast administration practices vary across practitioners and departments; however, standardised guiding principles and best practice recommendations can support improved infection control for radiology departments, educators, and policymakers aiming to enhance infection control within diagnostic imaging.

**Graphical Abstract:**

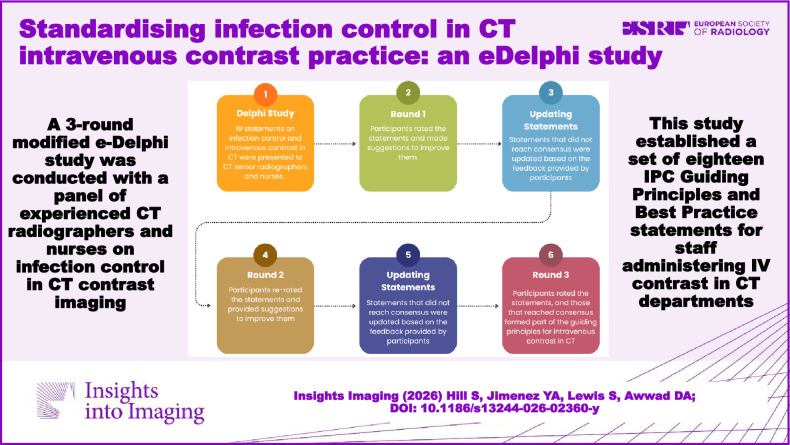

## Introduction

Computed tomography (CT) is the most common examination requiring contrast media worldwide. Safe administration of contrast requires strict adherence to infection, prevention and control (IPC), with guidelines and regulations imposed in clinical departments often at a state or national level, although a reasonable percentage of private radiology practices in Australia may not have infection control departments or officers [1]. Recent studies recognise that having clear policies and procedures around IPC practices in medical imaging departments is important for risk management [2–4]. Furthermore, effective IPC is essential in healthcare settings, yet adherence to IPC guidelines varies among radiographers in CT, impacting patient safety [4–6].

IPC for radiology, including user training, evaluation of aseptic techniques, contamination of fluid paths, and the use of single-use, multi-use and multi-patient devices, is under-explored in the literature [7]. Whilst technology is driving innovation, such as in the area of automatic contrast injection, evidence regarding contrast media use and best practice procedures in clinical settings is limited and does not recognise new practices with multi-use, multi-patient contrast administration systems [3]. Previous Australian studies have revealed scenarios where IPC compliance is lower, such as in emergency situations, yet an observational study has revealed that no two radiographers followed the same IPC steps during routine procedures among a cohort of 20 radiographers [3, 4]. Variations existed irrespective of whether single- or multi-use injectors were used, including if and when hand hygiene practices were followed, when gloves were worn or removed, and the order of steps to connect and disconnect the patient and contrast injectors [4].

Variations of practice and adherence to IPC practices within the CT suite have been documented, together with discrepancies in perceptions of risk and associated practice also noted [4, 5]. Additionally, radiographers are in close contact with many patients as radiology sees a high throughput of patients, with time pressures often cited as a cause of lower adherence to correct IPC practices [4]. Consequently, negative outcomes for patients have been reported for contrast-enhanced CT procedures, attributed to human error involving IPC through the use of contrast injectors. While national IPC guidelines exist, there is a lack of specific guidance tailored to CT IV contrast administration [1, 8]. Hence, this study aimed to develop consensus-based recommendations for IPC practices in CT.

## Methods

### Study design

A modified e-Delphi approach was used in this study [9]. The aim of the eDelphi method was to obtain group consensus regarding IPC in CT in the Australian healthcare setting. The multi-stage Delphi process allows iterative data collection from a group of experts regarding their opinion on a specific topic [9–11]. In this study, a modified e-Delphi technique provided an opportunity for a diverse panel of experts to be recruited from a variety of healthcare settings in Australia [9]. A total of three rounds were employed, involving anonymous online surveys consisting of predefined statements distributed to experienced CT radiographers and radiology nurses, who represented a panel of ‘experts’ due to their expertise in CT and use of contrast media in CT. Controlled feedback was provided by researchers after each round, including the percentage of agreement for each question, together with key points regarding the responses. All statements were either reposed or modified from the previous round.

This study was reported using the “Delphi studies in social and health sciences–recommendations for an interdisciplinary standardised reporting” (DELPHISTAR) reporting guidelines [12].

### Study recruitment

A survey invitation was sent to chief radiographers and advertised through the Australian Society of Medical Imaging and Radiation Therapy (ASMIRT) electronic newsletter targeting radiographers with five years or more of CT experience to participate in the study. The radiographers and nurses were deemed experts if they had self-reported a minimum of 5 years’ experience working in CT. The Research Electronic Data Capture software was used as the survey platform. The three rounds of surveys were undertaken from June 2024 to September 2024. The first round of the survey was open for four weeks. Subsequent round recruitment consisted of using the email addresses supplied by consenting participants in the preceding round, with participants able to opt out. Those who were not recruited or did not fit the participation criteria in the original round did not have an opportunity to join in later rounds. Subsequent rounds were open for four weeks, with a reminder email sent to participants two weeks after the initial email invitation was sent. The number of rounds was defined in advance to a minimum of two rounds, with the aim of being a maximum of three rounds. The third round would not have been conducted if there had been fewer than ten participants.

### Survey

The initial survey and subsequent rounds consisted of two sections. Firstly, participants were asked to provide information regarding their personal characteristics, education, CT experience, workplace and current IV contrast and power injector use as listed in Table 1. The survey consisted of 19 statements divided into two broad categories. The first category consisted of 4 statements, pertaining to Guiding Principles for IV Contrast. The second category consisted of 15 statements in relation to Best Practice for Use of IV Contrast. Each subsequent round of the survey contained the same sections and the same number of statements.

**Table 1:** Participant characteristics across three rounds of the modified eDelphi study

	Round 1		Round 2		Round 3	
	Number	Percentage	Number	Percentage	Number	Percentage
Age	Total = 34		Total = 26		Total = 24	
20–30	4	12%	3	12%	2	8%
31–40	17	50%	13	50%	13	54%
41–50	10	29%	9	35%	8	33%
51+	3	9%	1	4%	1	4%
Gender						
Woman	23	68%	17	65%	16	67%
Man	11	32%	9	35%	8	33%
Role						
Radiographer	31	91%	24	92%	22	92%
Nurse	3	9%	2	8%	2	8%
Education						
Undergraduate	11	32%	9	35%	10	42%
Postgraduate	7	21%	4	15%	2	8%
Postgraduate (grad cert, masters, etc)	15	44%	12	46%	11	46%
PhD	1	3%	1	4%	1	4%
Years of CT experience						
5–10 years	14	41%	6	23%	5	21%
10+ years	20	59%	20	77%	19	79%
Days/week in the CT department						
1	3	9%	5	19%	3	13%
2	4	12%	3	12%	2	8%
3	8	24%	6	23%	6	25%
4	5	15%	4	15%	4	17%
5	14	41%	8	31%	9	38%
Region						
Metropolitan	25	74%	19	73%	17	71%
Rural or remote	9	26%	7	27%	7	29%
Workplace						
Public hospital	26	76%	22	85%	22	92%
Private practice	3	9%	2	8%	1	4%
Private practice (in a private hospital)	3	9%	1	4%	0	0%
Private hospital	2	6%	1	4%	1	4%
Cannulate						
Yes	21	62%	11	42%	10	42%
No	13	38%	15	58%	14	58%

Statements were developed from various sources, including previous research [3–5, 7, 13–18] and National Health and Medical Research Council guidelines [1, 8, 19] based on concepts surrounding CT IV contrast injection and associated equipment, such as IV contrast power injectors. Approximately 50 statements were sourced by the research team, categorised and conceptualised. The researchers then reviewed statements for suitability. Statements that were out of the scope of radiographers' practice were removed from the study. Statements were reviewed and reworked to incorporate specific concepts, with those that held similar concepts combined to form one statement.

### Data collection, feedback and analysis

**Fig. 1:**
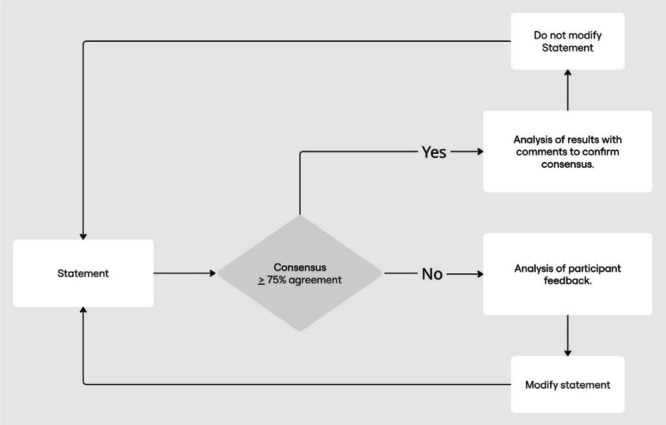
Summary of the modified eDelphi process used to reach consensus

Three eDelphi rounds were held with 19 statements posed each round. For each statement, participants selected a response on a 5-point Likert scale from ‘strongly agree’ to ‘strongly disagree’. Participants could also provide a justification regarding their selected response for each statement. Consensus was considered reached when 75% or more of the responses were either ‘strongly agree’ or ‘agree’ (Fig. 1). Dissent was considered when ‘strongly disagree’ and ‘disagree’ comprised 75% or more of the responses.

Statements that did not reach > 75% agreement in Round 1 and Round 2 were analysed by two independent reviewers and updated based on participants’ free-text responses, including instances where additional elements were suggested or adjustments were made to increase clarity (S.H. and D.A.A.). Hence, subsequent rounds of the survey were developed based on the results and feedback from the previous round. The landing page of the Round 2 and 3 surveys included a summary of the previous round’s aggregated response percentages and a description of modified statements. All statements that reached consensus in round 1 and round 2 were reposed in subsequent rounds to assess stability [9]. A statement was considered stable when consensus was reached and maintained across two consecutive rounds.

The participant characteristics were descriptively analysed. Agreement was calculated based on Likert Score, with ‘strongly agree’ and ‘agree’ statements calculated as a percentage of the total responses. Free-text analysis was conducted using thematic analysis based on posed statements.

### Ethical approval and consent to participate

Ethics approval was obtained from the University of Sydney’s Human Research Ethics Committee (Project number: 2022/493). All methods were carried out in accordance with relevant guidelines and regulations. Informed consent was obtained from all participants. The study was supported by funding from Imaxeon PTY LTD (Australia). The funding body had no role in the study design, data collection, analysis or manuscript preparation.

## Results

A total of three rounds were conducted, and there were 34, 26 and 24 participants per round, respectively. The participant characteristics are summarised in Table 1. Across the three rounds, more than 85% of participants were over the age of 30 years, with the majority of participants being female (average 67%). Of all the participants, approximately 50% had postgraduate qualifications across all rounds. There were 59%, 77% and 79% of participants who had over 10 years’ CT experience in each round, respectively. The mean number of days per week worked in CT in rounds 1, 2, and 3 was 3.68 (IQR = 2), 3.27 (IQR = 3), and 3.58 days (IQR = 2), respectively.

**Fig. 2:**
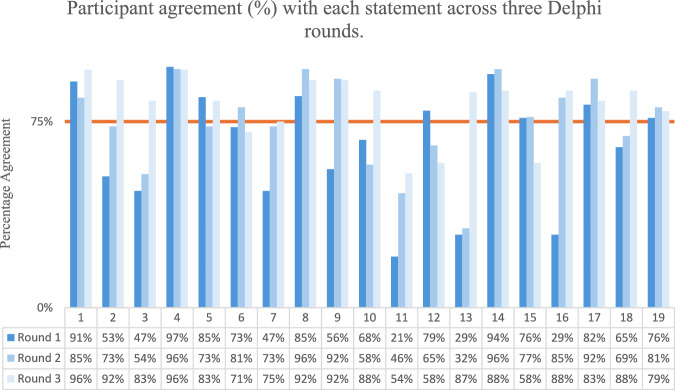
Participant agreement (%) with each statement across three Delphi rounds. Consensus threshold = 75% (orange line)

Following Round 1, nine statements reached consensus (Fig. 2). The remaining ten statements had 21%–73% agreement. These statements were modified based on participant comments and reposed in Round 2. Following Round 2, consensus was reached on a further three statements, but nine of the ten statements that did not reach consensus in Round 1 had improved in agreement (Fig. 2).

Statements posed in the final round are outlined in Table 2. All statements but Statement 11 reached consensus by the final round (Table 2 and Fig. 2). Overall, 42% (*n* = 8) of statements reached consensus and maintained stability throughout the three rounds (Fig. 2). The consensus was reached, but stability was not confirmed for 32% (*n* = 6) of statements due to consensus being reached in the final, third round (Fig. 2). Statements 6, 12, and 15 had initially reached consensus but the percentage of agreement decreased in subsequent rounds.

**Table 2:** Final-round consensus statements on guiding principles for IV contrast and best practices for the use of IV contrast

Guiding principles for IV contrast	
Statement 1	In addition to broad workplace IPC training, CT IV contrast-focused IPC education and training should be undertaken by staff.
Statement 2	Ideally, IPC training for CT IV contrast injectors, including informal refreshers and keeping current with technological updates, should be provided as changes are introduced.
Statement 3	Radiographers should undergo informal assessment (this could include audits, observations, summaries, etc) of the CT IV contrast injector to ensure continued compliance with IPC.
Statement 4	PPE should be available in key clinical areas of the CT suite, including close to patient contact areas, to promote IPC compliance.
Best practice for the use of IV contrast	
Statement 5	There are different IPC considerations between single- and multi-patient CT IV contrast delivery systems.
Statement 6	^a^While IPC principles remain consistent across different equipment, best practices for using CT IV contrast multi-patient delivery systems differ from those for single-patient delivery systems.
Statement 7	If best IPC practices are followed, both single- and multi-use CT IV contrast equipment offer equal risk for known infectious patients.
Statement 8	There is an ideal sequence when cannulating and/or connecting the CT IV contrast tubing to the patient.
Statement 9	Best practice when preparing the IV contrast injector between patients requires staff to use aseptic technique, including wearing gloves and/or hand hygiene.
Statement 10	It is possible to maintain IPC practices between the patient and IV contrast injector while applying different IPC techniques (e.g. hand hygiene, second radiographer, workflow).
Statement 11	^b^Dripped contrast/saline during or after connection to the patient is considered ‘contamination’ and therefore warrants changing gloves before touching the injector or other clean surfaces.
Statement 12	^a^Best practice for connecting the contrast tubing to the patient includes wearing gloves.
Statement 13	Replacement of the contrast tubing is required if disconnecting/reconnecting tubing to the same patient unless sterility is maintained.
Statement 14	Best practice indicates that gloves must be removed prior to leaving the CT scan room.
Statement 15	^a^Best practice for disconnecting the contrast tubing from the patient includes wearing gloves.
Statement 16	To disconnect the IV tubing between the patient and injector, a ‘clean’ to ‘dirty’ workflow is recommended.
Statement 17	For CT IV contrast equipment that is considered single-use, the syringe and associated equipment should NOT be reused/refilled for the same patient.
Statement 18	Best practice includes cleaning the CT IV contrast injector after every use unless it remains clean during a patient’s scan (e.g. hand hygiene before touching again after the patient).
Statement 19	Considering the unique CT suite environment, variations in good IPC practice are to be expected.

^a^ Statements losing stability after reaching consensus

^b^ Statements not reaching consensus by the final round

## Discussion

The aim of this study was to establish a set of IPC Guiding Principles and Best Practice statements for staff administering IV contrast in CT departments in the Australian healthcare setting. The use of an eDelphi approach was well suited to the Australian healthcare context, enabling participation from geographically dispersed radiology practitioners and supporting national representation across metropolitan, rural and remote settings. Facilitating asynchronous engagement, the eDelphi method allowed contributors to provide considered input without the logistical constraints of face-to-face consensus processes. Participants were provided with aggregated group responses between rounds to support iterative refinement of statements. Whilst this feedback is fundamental to Delphi methodology, it does carry the recognised risk of participants aligning responses with prevailing group views, risking independent judgement [11]. This potential influence should be considered when interpreting the robustness of the final consensus, particularly with statements whereby consensus could not be tested for stability.

The expert group reached consensus on the four ‘Guiding Principles for IV Contrast and Compliance’ statements by the completion of the study. However, only 14 of the 15 ‘Best Practice for use of IV Contrast’ statements reached consensus, with Statement 11 not reaching consensus by the completion of the study. Furthermore, the study assessed statements for consistency across each round (stability), of which those that reached consensus in earlier rounds remained stable, except statements 6, 12, and 15.

Statements on the guiding principles of IPC (Statements 1–4) indicate there is awareness and recognition that training and compliance regarding IV contrast and associated equipment are important and should be included as part of initial IPC training and to ensure continued compliance with changes to technology [16]. These statements recognise that adherence to IPC is promoted when access to personal protective equipment (PPE) is readily available for healthcare workers in the department and are similar to the findings of other studies [5, 20].

Participants indicated that there are different IPC considerations between single- and multi-patient CT IV contrast delivery systems, with best practices differing between the delivery systems. This approach to the different systems is consistent with the steps required for the preparation and delivery of various CT IV contrast systems outlined by Toia, G.V. et al and confirmed with observational studies performed on CT IV contrast administration [3, 21]. Participants of this study indicated that while there are variations in sequencing when using IV contrast injectors, IPC is not compromised by these variations in practice, which is consistent with recent studies that indicate sequencing is highly varied amongst health care workers [3].

Statement 8 explored whether an ideal sequence exists when cannulating and/or connecting CT intravenous contrast tubing. Free-text responses highlighted two complementary themes. First, participants emphasised that adherence to an ordered sequence is important for maintaining IPC practices and minimising infection risk during contrast administration. Second, respondents noted that sequencing must remain adaptable to accommodate patient-specific factors, including anatomical challenges, clinical urgency, and patient comfort. These responses indicate expert agreement that structured sequencing plays a key role in reducing IPC-related risk, while also recognising the need for contextual flexibility in real-world clinical practice. This balance between standardisation and clinical adaptability has implications for guideline development, suggesting that IPC recommendations should define core sequencing principles while allowing discretion in response to individual patient circumstances.

The statement regarding dripped contrast (Statement 11) did not reach consensus by the final round, with participant feedback relating to the perception of risk surrounding what is touched and when. This is consistent with other studies that report that this perception of risk drives decision-making surrounding the use of gloves for procedures [3, 17, 20]. Interestingly, agreement regarding contamination from dripped contrast doubled when the statement was amended to specify that the contamination occurred during or after connection to the patient; however, it still failed to reach the 75% consensus threshold by the final round, indicating there is still some dissonance in the perception of risk and how this is applied in practice.

Statements 12 and 15 considered the process of connecting and disconnecting the contrast tubing to the patient’s bung with gloves. Both statements were considered unstable and lost consensus over the rounds as participants provided comments surrounding perception of risk or their understanding of the policy requirements surrounding aseptic techniques for the administration of IV contrast. Whilst participants commented that hand hygiene was sufficient for this particular process, the researchers agreed to continue to provide the same statement across all three rounds, specifically using gloves because it was consistent with the current National Health and Medical Research Council guidelines regarding the access of peripheral IV cannulas [1, 19]. Previous studies observed that radiographers have a strong knowledge of what is considered the key parts of IV contrast equipment and emphasised not touching sterile components irrespective of whether gloves are worn [3, 22]. Using discretion regarding IPC considerations when preparing the IV contrast injector allows for the use of hand hygiene alone or hand hygiene with gloves [3]. This aligns with National Health and Medical Research Council guidelines suggesting that, where asepsis of gloved hands can be maintained between preparation and administration, additional hand decontamination may not be required, thereby still maintaining IPC compliance but allowing workflow efficiency [19]. However, there are additional risks when it comes to bungs in which IPC guidelines suggest that “*Gloves should be worn as part of standard precautions when there is a risk of contact with blood, body fluids or surfaces and equipment contaminated by infectious agents”* [8]. In regard to connecting and disconnecting contrast tubing to and from the patient, participants indicated that their view of the risk of contact is particularly nuanced. Given the similar terminologies “key parts” and “aseptic technique” together with the proximity of the steps within the procedure, it is understandable that participants in this study concluded that gloves are not required and could be considered a hindrance to maintaining IPC, especially when hand hygiene is sufficient in other parts of the overall process of IV contrast administration. The results from this study are echoed in previous studies that demonstrated radiographers' understanding that by not touching key parts, they are maintaining IPC aseptic techniques [21].

## Limitations

This study was conducted within the Australian healthcare context, using Australian national guidelines, which may limit applicability to other healthcare systems. Nurses were underrepresented compared with radiographers, and no nurses from private institutions completed the study, limiting the generalisability of nursing-specific findings. Most participants were based in metropolitan settings and public hospitals, where formal IPC teams are typically present. This panel composition may have contributed to the relative homogeneity of perspectives, noting that greater heterogeneity can strengthen the robustness of Delphi consensus. No infectious diseases specialists were included, as recruitment was limited to CT-focused clinicians; inclusion of such expertise in future studies may further strengthen the contextual validity of the recommendations. In addition, data on participants’ formal IPC training or dedicated IPC roles were not collected, limiting assessment of the influence of specialist IPC expertise on the consensus. Nonetheless, panel members contributed substantial practical experience in CT contrast workflows where IPC principles are routinely applied in Australian clinical practice. Another key limitation is the attrition between the three eDelphi rounds, starting with 34 participants and ending with 24. While most demographic characteristics remained stable across the three rounds, there was a higher proportion of more experienced participants based in public hospitals and a lower proportion of participants who could cannulate by the final round. This shift may reflect differential engagement of senior clinicians or variations in scope of practice across settings, particularly in public hospitals where there is a higher proportion of nurses in CT compared with the private sector [15]. While attrition is an expected feature of Delphi studies, it is important to acknowledge that loss of participants can reduce the diversity of perspectives represented within the panel, which may have contributed to greater convergence of opinions and increased likelihood of consensus in the later rounds. Nonetheless, as IV cannulation does not encompass all CT-related IPC practices, and many IPC-relevant steps are performed by both canulating and non-canulating practitioners, this change does not necessarily diminish the relevance of the consensus to CT contrast IPC more broadly.

## Conclusion

This study established a set of eighteen IPC Guiding Principles and Best Practice statements for staff administering IV contrast in CT departments in the Australian healthcare setting. Given the variability in contrast administration processes among radiology practitioners and departments, this eDelphi study systematically elicited and refined expert opinion through iterative rounds to achieve consensus on IPC practices, producing transparent and reproducible outputs to support standardisation and inform policy development. Recommendations can also be used to inform future IPC education for radiology staff, with IPC considerations consistently underpinning decision-making surrounding these processes.

## Data Availability

All authors had full access to all of the data, including statistical reports and tables related to the study. The data for this study will not be shared, as we do not have permission from the participants or ethics approval to do so.

## Abbreviations

CT: Computed tomography
DELPHISTAR: Delphi studies in social and health sciences–recommendations for an interdisciplinary standardised reporting
IPC: Infection, prevention and control
IV: Intravenous
